# Supplementary figures and images for: Isoferulic acid facilitates effective clearance of hypervirulent Klebsiella pneumoniae through targeting capsule
Source: PLoS Pathog. 2025 Jan 6;21(1):e1012787. doi: 10.1371/journal.ppat.1012787 (PMC11737856; doi:10.1371/journal.ppat.1012787)

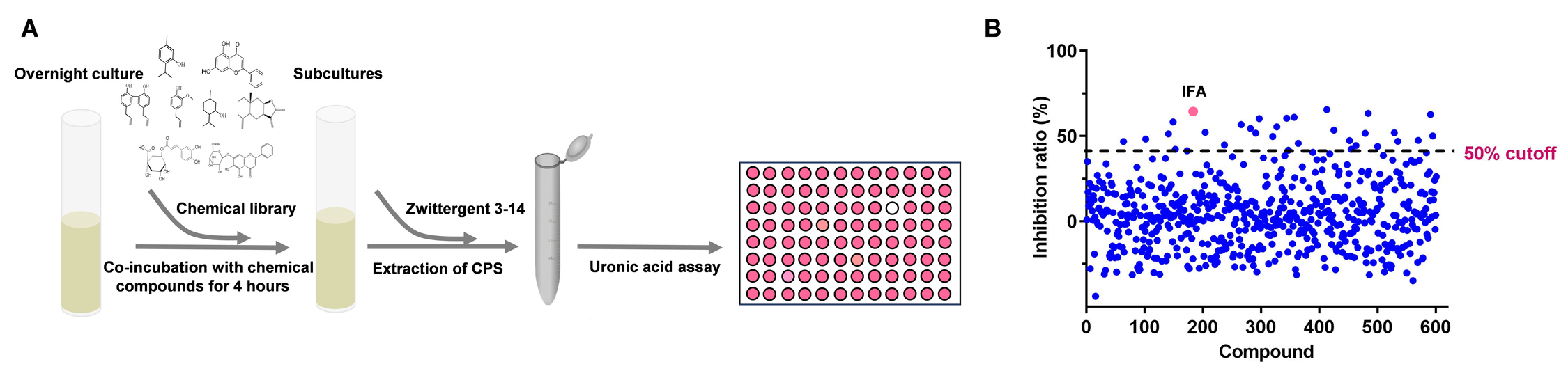

Supplement: S1 Fig — (A) Diagram for compound screening of capsule inhibitors. (B) Percentage inhibition of uronic acid levels by each compound tested at 32 μg/ml. (TIF) [file ppat.1012787.s001.tif]

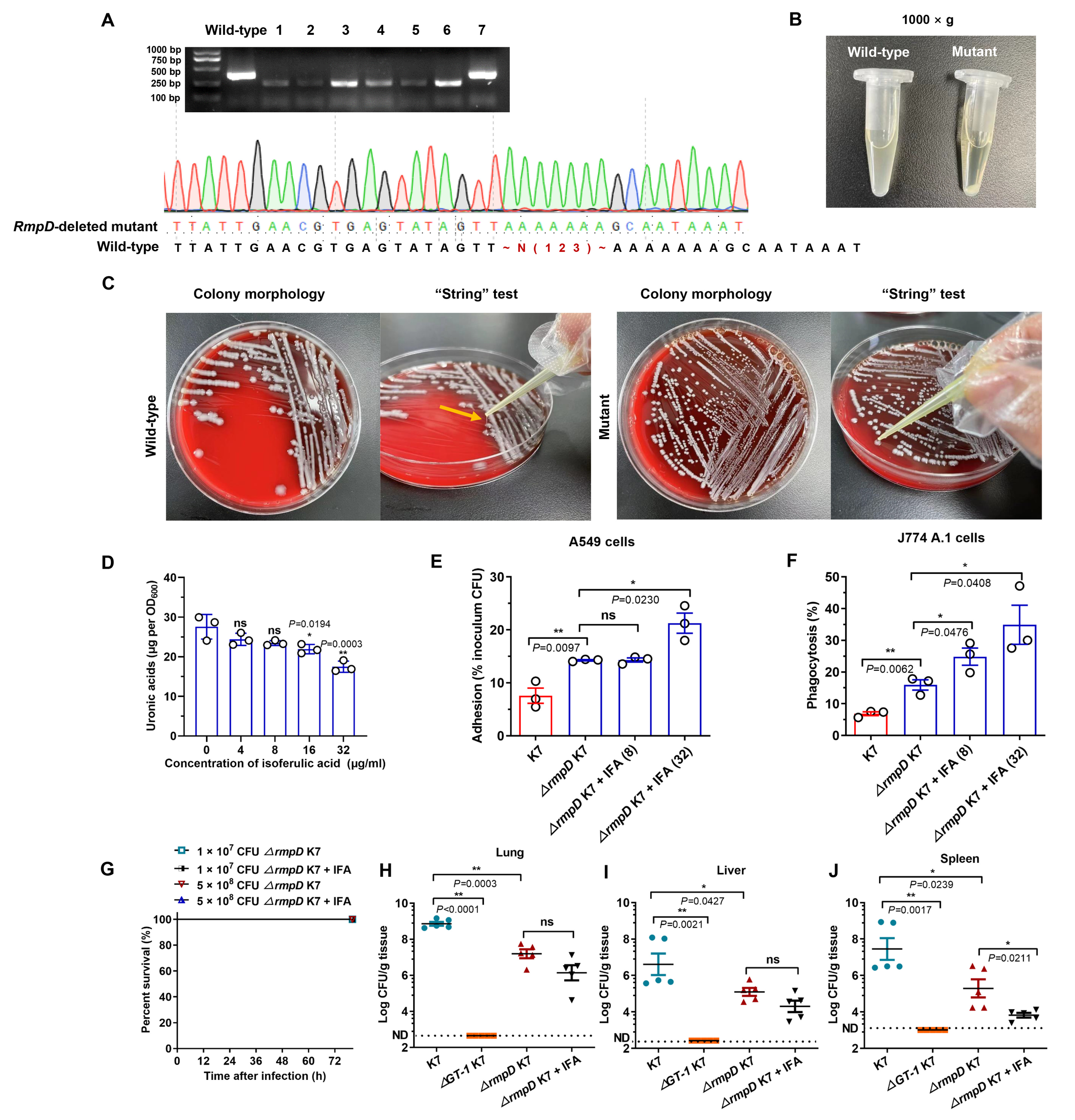

Supplement: S2 Fig — (A) The deletion of the rmpD gene in K7 strain. The PCR band from wild type strain served as a positive control. (B) The semiquantitative sedimentation assay of wild type strain and ΔrmpD-K7 mutant. (C) The colony morphology observation and “string test”. (D) Determination of capsule production by uronic acid assay. Total uronic acid samples were extracted from ΔrmpD-K7 mutant co-cultured with indicated concentrations of IFA. (E) Adhesion assay of K. pneumoniae K7 and ΔrmpD mutant to A549 cells in the presence of DMSO or indicated concentrations of IFA. Cells were infected with K. pneumoniae strains at an MOI of 50 for 2 hours and then washed, lysed and plated on LB agar plates to quantify the colony-forming units. Data are presented as a percentage of the initial inoculum CFU. (F) Phagocytosis of K. pneumoniae K7 and ΔrmpD mutant by J774 macrophages in the presence of vehicle or indicated concentrations of IFA. Cells were infected at an MOI of 5 for 2 hours and then washed, followed by further 1-hour incubation in the medium containing gentamicin (100 μg/ml) to kill extracellular bacteria. The cells were then rinsed, lysed and plated on LB agar plates after serial dilution. (G) Survival of mice challenged by 1 × 10^7^ (n = 6 mice each group) or 5 × 10^8^ ΔrmpD K7 (n = 8 mice each group) with indicated treatments. Mice were subcutaneously injected with 50 mg/kg IFA in 50 μl of 10% DMSO containing vehicle (10% DMSO, 45% stroke-physiological saline solution, 40% PEG400 and 5% Tween-80) or an equal volume of vehicle immediately after infection, and the number of deaths was recorded for survival analysis. (H) Bacterial burden in the lung tissues of mice infected with K. pneumoniae strains. Mice challenged with 5 × 10^7^ wild-type K7, ΔGT-1 K7 and ΔrmpD K7 bacteria were treated as indicated and sacrificed at 40 hours post infection, and lung tissues were removed and homogenized in PBS to analyze the bacterial burden by microbiological plating (n = 5 mice each group). The bacte [file ppat.1012787.s002.tif]

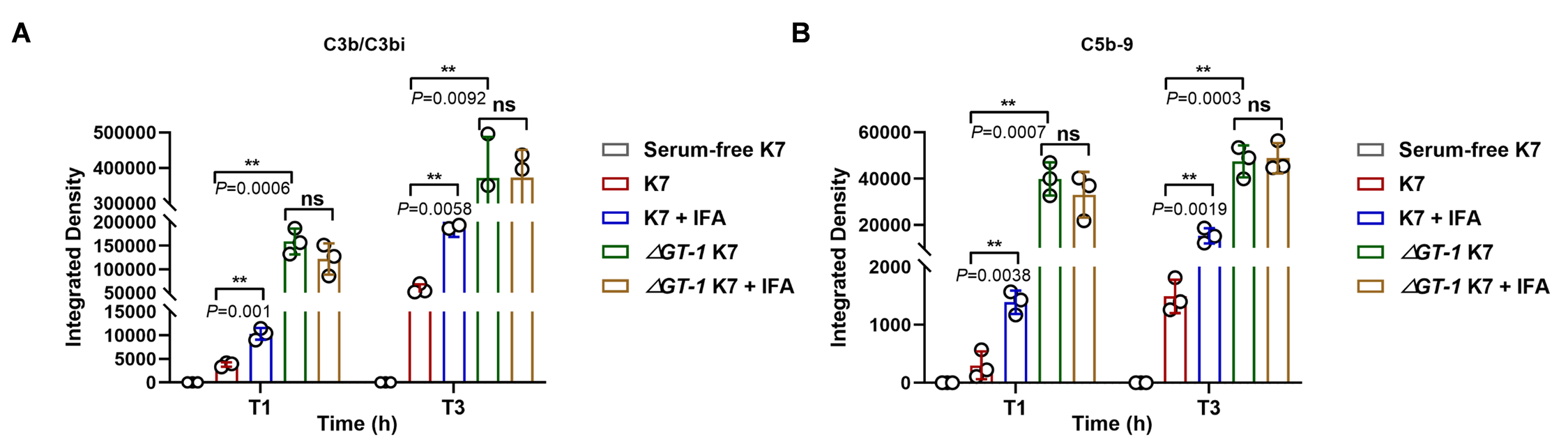

Supplement: S3 Fig — (A) The immunofluorescence intensity of C3b/C3bi quantified by Image J. (B) The immunofluorescence intensity of C5b-9 quantified by Image J. The mean ± SEM is shown. Data were analyzed using unpaired two-tailed Student’s t-test. **P < 0.01 and ns, no significance. (TIF) [file ppat.1012787.s003.tif]

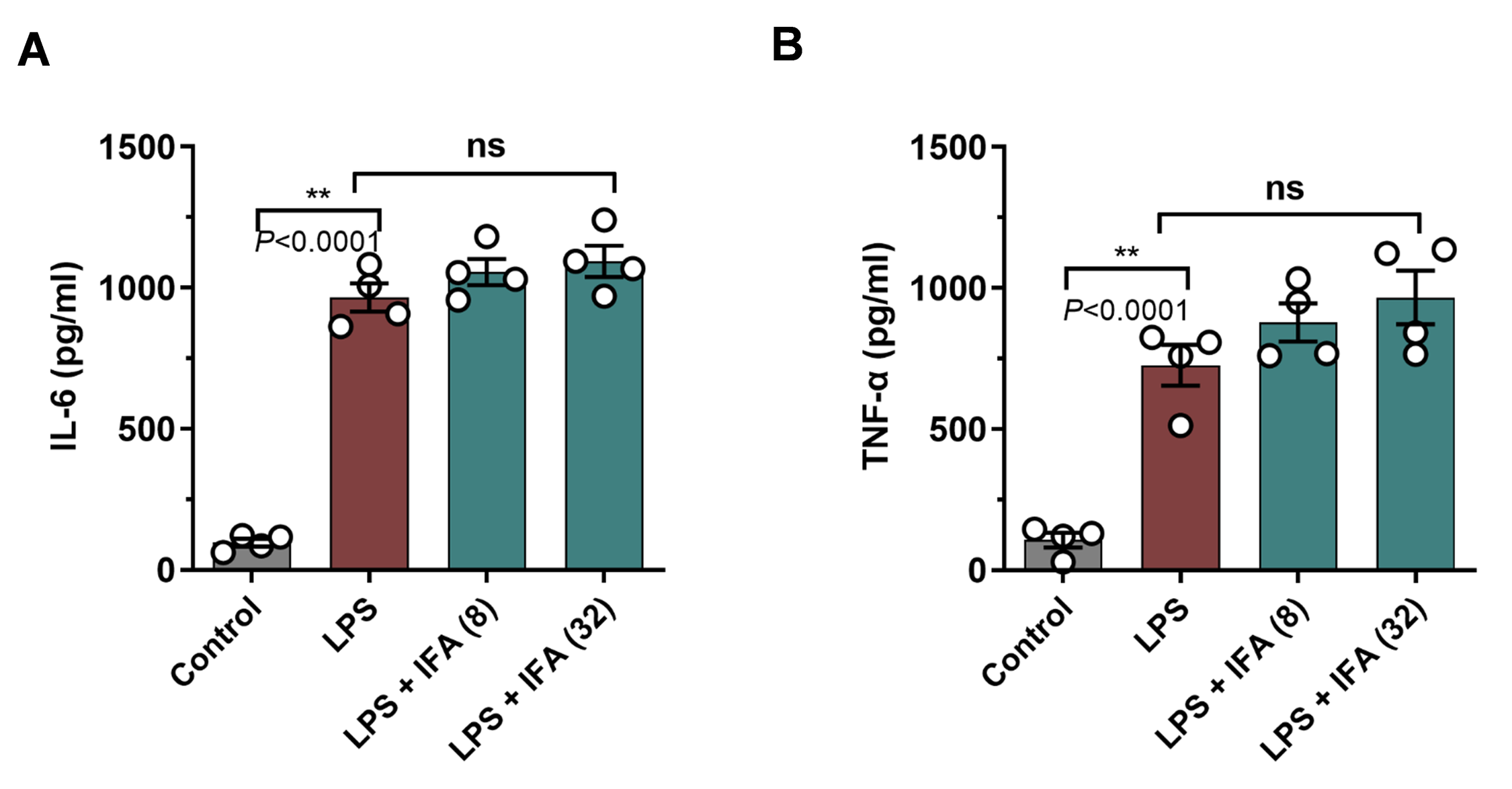

Supplement: S4 Fig — To determine the effect of IFA on the TLR4 ligand LPS-stimulated inflammation response, mouse primary peritoneal macrophages (MPMs) were stimulated with LPS (1 mg/mL) for 18 hours in the presence of DMSO or the indicated concentrations of IFA, and the levels of IL-6 (A) and TNF-α (B) in the culture supernatants were detected using ELISA. The data are presented as the means ± SEMs. Data were analyzed by one-way ANOVA and Tukey’s posttest. **P < 0.01 and ns, no significance compared with DMSO control. (TIF) [file ppat.1012787.s004.tif]
