# Supplementary material for: Isoferulic acid facilitates effective clearance of hypervirulent Klebsiella pneumoniae through targeting capsule
Source: PLoS Pathog. 2025 Jan 6;21(1):e1012787. doi: 10.1371/journal.ppat.1012787 (PMC11737856; doi:10.1371/journal.ppat.1012787)
Supplement: S1 Table — (DOCX) [file ppat.1012787.s005.docx]

Table 1 Sequence of primer pairs used in the study.

| Gene | Primer | Sequence (5´-3´) |
| --- | --- | --- |
| *p*CasKP-apr  detection | Sense | ggtgttacatgctgttcatctgttacattgt |
|  | Anti-sense | cgattcttccgacgtgtataccttctac |
| *p*SGKP-spacer-spec  detection | Anti-sense | caggaaacagctatgacc |
| *rmpD* spacer | Sense | TAGTtcatcgcttttctgcgcgagcgg |
|  | Anti-sense | AAACccgctcgcgcagaaaagcgatga |
| *rmpD-*up | Sense | tatcgaattcgattttaaaataagtcctaaactcg |
|  | Anti-sense | gaacgtgagtatagttaaaaaaagcaataaataaataaaag |
| *rmpD-*down | Sense | ttattgctttttttaactatactcacgttc |
|  | Anti-sense | tagtggatccataatggagcggaaaataaaaatctcatccc |
| *rmpD-seq1* | Sense | tacagccttaataaagaaatggttg |
|  | Anti-sense | atgctttgacatatttgatagatgtt |
| *rmpD-seq2* | Sense | aatgggggcattaatgaaataaaaag |
|  | Anti-sense | atccttctttctttatatgttctcatttg |
| *gnd* | Sense | taaccgtgaactgtctgcag |
|  | Anti-sense | ccgatataagtcacacacgg |
| *wcaJ* | Sense | gtgtgtgttttcagtggggttg |
|  | Anti-sense | gatggcgtaagtgatactataccc |
| *wbaP* | Sense | ctctcggcatgctgtcatttac |
|  | Anti-sense | ccattttgcaaaggccattattgc |
| *wzx* | Sense | gttggcaacgttcagatggc |
|  | Anti-sense | gcagaaatggaagtgcaacca |
| *wza* | Sense | ccggaattaacgacacctg |
|  | Anti-sense | gggctttcaatatatgtggttaatc |
| *wzb* | Sense | cctataaggatcaggaatatctctttg |
|  | Anti-sense | ggtcatttaggaagacaatttacatc |
| *rcsB* | Sense | ccatccgatcgtactgttcg |
|  | Anti-sense | cagcgtgatcccgtcgc |
| *rfaH* | Sense | caggaacatctggaacgtcagtcag |
|  | Anti-sense | cccttccggtttgtagatagaaag |
| *rmpA* | Sense | gacagcaggattttttattcaggg |
|  | Anti-sense | catttccttgcatgttaacatagatg |
| *rmpC* | Sense | gaacacaaactgtaatgttaattcc |
|  | Anti-sense | ggctgttttttcacttatctgtg |
| *rmpD* | Sense | ctataatagtttgaatttttttttgcg |
|  | Anti-sense | ttgttttttttgtcagtttataattc |
