# Supplementary material for: Isoferulic acid facilitates effective clearance of hypervirulent Klebsiella pneumoniae through targeting capsule
Source: PLoS Pathog. 2025 Jan 6;21(1):e1012787. doi: 10.1371/journal.ppat.1012787 (PMC11737856; doi:10.1371/journal.ppat.1012787)
Supplement: Attachment — Submitted filename: Point to point response.docx [file ppat.1012787.s006.docx]

**Point-by-point responses to the Editor and expert referees**

**Response to the editors**

Dear Dr. Michael Malim, Dr. D. Scott Samuels and Dr. M Ammar Zafar, thank you very much for your kind letter and your careful work regarding our manuscript (PPATHOGENS-D-24-01091). In addition, we would like to express our sincere gratitude for providing us with sufficient extension to prepare the revision. We have addressed your concerns about the redundancy of the manuscript, the potential effect of IFA on *rmpD-*deficient mutant, the inappropriate citations, the concerns regarding IFA as vehicle control, and the screening procedure identifying IFA as a capsule inhibitor. The manuscript has been revised in accordance with the reviewer’s comments by performing additional experiments and modifying the text and figures. We sincerely appreciate the comments that you and the reviewers provided, with these valuable suggestions, the revised manuscript has been significantly improved, and they also provide much help for our future work. We hope that this revised manuscript has met the editorial requirements you and the reviewers described. Your attention to our revised manuscript is sincerely appreciated. The following are our responses to the reviewer’s comments.

**Response to Reviewers**

We would like to thank the reviewers for the positive and constructive comments, which have essentially improved our manuscript.

**Part I - Summary**

**Reviewer #1**

In this study by Wang and colleagues, the authors identify the compound isoferulic acid (IFA) as an inhibitor of Klebsiella pneumoniae capsule production. Using a combination of in vitro and in vivo techniques, the authors define IFA inhibition of K. pneumoniae capsule and seek to determine how this inhibition influences bacterial pathogenicity and host responses. One of the great strengths of this study is the utilization of several techniques to confirm early key findings. For example, uronic acid quantification, SEM, centrifugation assays, and the string test are all used to characterize and differentiate capsule and hypermucoviscosity. While the initial characterization of IFA in capsule inhibition is quite interesting, I have major concerns about much of the characterization of IFA in host immune responses. In many instances statistical comparisons are made between irrelevant groups, and some data implies that the IFA vehicle itself is modulating host responses. Additionally, the influence of K. pneumoniae capsule on host immune responses has been previously extensively characterized and I am not convinced that this study is providing novel insight into capsule biology.

**Response:** We greatly appreciate your positive comments on our manuscript.

As you mentioned, the interaction between capsule and host immune response including capsule-specific engagement of TLR4 pathways has been well studied. In the present study, we chose initially to further confirm that IFA indeed accelerates host immune clearance of *K. pneumoniae* through inhibition of capsule, rather than illustrating capsule in innate immune responses, or capsule-specific engagement of TLR4 pathways. However, the vast number of experiments have made it exhausting and confusing to follow. To make the manuscript more readable, we removed experiments on TLR4-mediated immune responses and epithelial defensins according to your professional suggestion and the comments from Reviewer #3. Notably, we remained the phagocytosis and adhesion experiments, both of which are classical resulting phenotypes characterizing capsule-mediated immune evasion in the field of *K. pneumoniae*.

We completely agree with your concern about whether IFA itself modulates host immune response. Although we have deleted the relevant data, we further detected the expression of autophagy-related genes and type I IFN genes in mouse peritoneal macrophages that were treated with indicated concentrations of IFA for 6 hours without *K. pneumoniae* infection according to your suggestion. As shown below, IFA did not exhibit visible effect on the transcription levels of those genes.

Furthermore, we have carefully checked all the statistical comparisons shown in the results and removed the irrelevant comparisons.

Please refer to the point-to-point reply below. We hope that this revised manuscript meets the requirements you described.

**Reviewer #2**

This manuscript by Wang, Yang et al. reports the findings of a study exploring the effects of isoferulic acid (IFA) on Klebsiella pneumoniae (Kp) capsule expression and resulting phenotypes. The authors should be commended for their extensive experimental approach. This greatly increases the confidence in the authors' conclusion that IFA represses capsule to a degree that impacts the pathogenesis of Kp. Given that the role of capsule in Kp virulence is very well studied, much of data are supporting, rather than discovery; however, this does increase the confidence in the conclusions regarding the impact of IFA on Kp. Unfortunately, the in vivo regiment for IFA delivery is very intense (3 injections/day), potentially hindering the potential of IFA as a therapeutic. Thus, the authors conclusions regarding the therapeutic use of IFA may be overstated. There are many areas where the manuscript can be clarified, and it would benefit from another round of careful editing.

**Response:** Thank you for your positive comments and careful review. We have revised the overstated conclusions throughout the manuscript according to your suggestion. Please see lines 40-42 and 58-59.

Furthermore, all the mis-labeled and insufficient panel labels, overstated conclusions and other detailed problems throughout the manuscript have been carefully revised according to your careful review.

Please refer to the point-to-point reply below. We hope that this revised manuscript meets the requirements you described.

**Reviewer #3**

Wang, et al. (PPATHOGENS-D-24-01091) report a series of experiments showing that treatment of hypervirulent Klebsiella pneumoniae cultures with a newly-identified compound called IFA reduces capsule and hypermucoviscosity (HMV). These phenotypic changes render the bacteria more sensitive to complement binding and killing, less virulent in both Galleria and mouse models of infection, and altered immunological responses by the host. Given the growing threat of antibiotic resistant Klebsiella, new therapeutics are much needed and the data presented here have value to the community. The strengths of this manuscript lie in the vast number of experiments showing that IFA treatment improves host survival, most likely by reducing capsule production/HMV. The organization of the data is logical and the experiments seem to be well-executed with appropriate controls. However, the vast number of experiments is also a weakness! I found myself exhausted by the volume of data and by the challenges of examining many small data panels that were a lack of clarity with some experiments. Most comments provided below are in an effort to make this manuscript more readable.

**Response:** Thanks a lot for your positive comments and constructive suggestions. We have deleted some redundant data to make the manuscript more readable, including the experiments on TLR4-mediated immune responses and epithelial defensins, and some unnecessary statistical analyses, such as those shown in Figure 2E.

In addition, we have successfully constructed a *rmpD*-deficient mutant and performed in vitro and in vivo experiments to clarify if IFA indeed suppressed HMV through targeting capsule rather than the HMV regulator RmpD, and also to confirm whether its protection against hvKP infection was related to capsule itself, HMV or both. All the mis-labeled and insufficient panel labels, overstated conclusions and other detailed problems throughout the manuscript have been revised according to your careful review.

Please refer to the point-to-point reply below. We hope that the revised manuscript has met your requirements.

**Part II – Major Issues**

**Reviewer #1:**

1.The field of Klebsiella pathogenesis has extensively characterized the impact of capsule in pathogenicity and the authors state that capsule is one of K. pneumoniae’s most relevant virulence factors. For example, in previous studies authors have determined capsule to be important for in vivo virulence (PMID16262790), serum susceptibility (PMID26060277), and TLR4-mediated inflammation (PMID25971969). These references, and many others, have extensively established that K. pneumoniae capsule helps to protect the bacteria from innate immune threats. I am highly enthusiastic about the author’s findings in Figures 1-3, but am not convinced that the authors have uncovered novel biology in latter figures as these results largely confirm previous findings. The study would be stronger if the paper’s focus was solely the suppression of capsule by IFA in Figures 1-3, and not focused on the role of capsule in pathogenicity.

**Response:** We agree with and appreciate your comment. According to your suggestions and those of the other reviewers, we have removed some redundant data to make the manuscript more readable, including the experiments on TLR4-medated immune responses and epithelial defensins, and some unnecessary statistical analyses, such as those shown in Figure 2E. Please see the new figures and revised manuscript.

2.The statistical comparisons in the latter part of the manuscript are not always appropriate for making the conclusion that IFA-mediated inhibition of K7 capsule is modulating the immune response. In many panels, the authors neglect to show statistical differences between the WT and acapsular K7 strains (Figure 4F, 4H). This is important for first demonstrating that lack of capsule is important for the methodology, then determining that a WT+IFA comparison has a similar result. In Figure 5A&5B, a comparison between K7 and acapsular K7 would likely show no difference in expression of the gene is interest, so it is curious why the IFA administration significantly increases expression in many cases. The authors report these comparisons in 5C&5D, the approach should be consistent in other panels throughout the manuscript.

**Response:** We agree with and appreciate your comment.

(1) We have supplemented the statistical comparison between WT and acapsular K7 strains throughout the text, including Figure 4F and 4H, and the necessary result statements were also added to the manuscript as you suggested, which makes our results more rigorous. Please see the revised figure 4 and lines 213-215, 223-225, 237-240, 257-258, 267-272 and 280-282.

(2) Although, we removed the experiments on TLR4-mediated immune responses and epithelial defensins according to the suggestions from you and editors, as well as reviewer #3, we further detected the expression of autophagy-related genes and type I IFN genes in mice peritoneal macrophages that were treated with indicated concentrations of IFA for 6 hours without *K. pneumoniae* infection. As shown below, the results suggested that IFA did not up-regulated type I IFN signaling and autophagy-related genes. Thus, we hypothesized that although the thickness of *K. pneumoniae* capsule was strongly reduced by IFA, the remaining capsule structure could still be recognized by TLR4 and was sufficient to activate TLR4-mediated IFN signaling and autophagy response. Moreover, IFA enhanced the phagocytosis of wild-type *K. pneumoniae,* but not acapsular K7, which confirmed our speculation and partly explained why IFA boosted the activation of these bacterial clearance-promoting immune responses.

3. Perhaps my biggest concern is the possibility that the IFA vehicle is substantially modulating immune responses. For example, in Figure 5 there are many panels in which the WT and acapsular K7 strains elicit similar expression of the genes of interest. However, there is a substantial increase in expression of certain genes when IFA is added (ex. Figure 5A, B, F). Again, in Figure 7D the authors claim that acapsular K. pneumoniae elicits less LDH release than the WT strains (Lines 333-334) but this statistical comparison is not made in the corresponding graph. By eye, it appears that the WT and acapsular K7 strains elicit quite similar LDH release. Since the statistical comparison is then significant between the K7 and the K&+IFA strains, this implies that the IFA vehicle could be influencing LDH release. To underscore this point, the control in the LDH experiment is a PBS control, not the vehicle in which IFA is administered. Another example can be found in Figure 6B, where there is a substantial change in defensin1 expression when the acapsular K7 is treated with IFA. Another instance of potential vehicle effects is in animal modeling. In Figure 7C, the Galleria treated with K7+IFA have substantially higher OD490 than the K7 or the acapsular strains.

**Response:** Thanks for your patient recommendation.

(1) Addressed as described in the above response to Q2, we have removed the experiments on TLR4-mediated immune responses and epithelial defensins to make the story more readable according to your suggestion. Please see the response to Q2 and our revised text.

(2) We really appreciate your insightful evaluation on the LDH release assay. As you mentioned, the insignificant difference in LDH release between WT and acapsular K7 group might be attributed to the administration of the IFA vehicle, which contains 10% DMSO. Thus, we carried out the experiment again and reduced DMSO to 1%, and the PBS control was also treated with IFA vehicle. As shown in the panel below (new Fig 6D), the level of LDH release was greater in the wild-type K7 group than in the other groups, including the acapsular mutant and K7+IFA group. Notably, necessary statistical comparisons and results statements were also added to the new figures and text. Please see lines 886-887.

(3) We apologize for the mislabel in Figure 7C. K7 and K7+IFA were mis-labeled in this panel, and the mistake has been corrected in the new Fig. 6C.

4. The authors do an excellent job of defining IFA as a compound that suppresses capsule, but the study itself should be streamlined to emphasize the most important points. It is unclear whether the authors view their major finding as the identification of IFA-capsule interactions, characterizing capsule in innate immune responses, or capsule-specific engagement of TLR4 pathways. The beginning of the manuscript has clear rationale (Figures 1-3), but the story becomes convoluted in the latter parts (Figures 4-10). The authors should streamline the text to justify jumping between epithelial cells and macrophages, two different in vivo models, and explanations of why genes of interest in the expression data are important.

**Response:** Thank you again for your scientific and professional comments. Our story focused mainly on defining IFA as a capsule inhibitor that renders hvKP more sensitive to complement killing and host cell clearance and therefore, less virulent to host, which would help develop new strategies for curbing resistant *Klebsiella* infections. However, the manuscript was truly weighed down by the experiments on the mechanism how IFA accelerated bacterial clearance through interfering the interaction between capsule and host immune response. As you suggested, removing unnecessary results is a very necessary modification. Thus, we have deleted some redundant parts to make the highlights of our article clearer and more readable, including TLR4-mediated response and defensins expressions as described above. Please see the new figure 5 and lines 231-252, 861-872.

**Reviewer #2:**

1. The authors indicate that the IFA was identified as part of a larger natural compound screen, yet no data or citation is provided for this screen and its conditions. This may be important context for the approach and valuable information for the field.

**Response:** We agree with and appreciate your comment. According to your suggestion, we have added a screening procedure and the initial screen top hits as S1 Fig and detailed them in the manuscript. Please see the updated figures and lines 135 and 758-760.

2. The data concerning autophagy markers is quite confusing. Some markers in the IFA treated group are equal to or lower than that of the untreated control, whereas others are significantly increased compared to untreated and treated controls. Without a more thorough exploration of autophagy, it is difficult to draw high-confidence conclusions from these data, especially compared to the clarity of the other TLR4-mediates phenotypes.

**Response:** Thank you for your professional suggestion. In accordance with the suggestions of the editors, Reviewer #1 and Reviewer #3, we have removed some redundant data to make the manuscript more readable, including the experiments on TLR4-mediated type I IFN signaling and autophagy response, as well as the detection of defensins levels in A549 cells. Please see the revised Figure 5 and lines 231-252, 861-872.

3. Many of the citations in the manuscript are inappropriate or unclear. The authors should review their references and ensure they are citing relevant literature. Examples include:

a. Lines 75-78 - the referenced citation is not appropriate for this statement. Mike et al. systematically demonstrates linkages between capsule and hypermucoviscosity. This statement should be supported by primary literature or an appropriate primary literature review.

b. Lines 107-109 - the referenced citation is not appropriate for this statement. Ernst et al. specifically interrogates ST258 Kp, rather than hvKp.

c. Lines 111-114 - statements need citations.

d. Line 269 - citation 29 is not related to autophagy.

e. Line 281 - this review does not mention Kp.

f. Line 319, 332 - these are single studies; however, the authors' statement reflects the state of the field. This is not an appropriate way to support these statements.

Response: We appreciate your careful review. We have revised those inappropriate citations you listed and carefully checked this issue throughout the manuscript. Some citations are not included because of the removement of TLR4-mediated immune response and defensins expressions. Please see lines 78, 101-102 and 104-108.

**Reviewer #3:**

1, This is an extremely large collection of experiments that I found somewhat counterproductive. One broad suggestion is to choose 2-4 key experiments per figure and either delete or move the rest to supplemental data. Figs 1 and 2 would be much more digestible if ~4 experiments were chosen and the rest removed due to their citat.

**Response:** We agree with and appreciate your scientific and professional comments. According to the suggestion of you and the editors, we have removed some redundant data to make the manuscript more streamlined and readable, especially the experiments for clarifying the effect of IFA on capsule-host interactions. Please see the revised Fig 5 and lines 231-252, 861-872.

2, Many conclusions are overstated. For example, from Fig 1, the authors conclude that the reduction in CPS following IFA treatment is due to inhibition of CPS synthesis. It could be synthesis due to reduced enzymatic activity OR changes in cps or rmp gene expression. Alternatively, it could be that export is affected. From Fig 2, they state that HMV is reduced due to hindered CPS synthesis. While this could be true, they have not drawn a direct connection. There are reports of Klebsiella strains that retain the HMV phenotype even though CPS is reduced. Downregulation of the rmp genes could also lead to this phenotype. I have similar concerns over the frequent statements that IFA reduced capsule chain length based on Fig 1D (see details in comment below).

**Response:** Thanks for your comments. We have carefully checked the issue you raised and revised all the overstated conclusions, especially those in Fig 1, Fig 2. Regarding the concerns on HMV, we detected the transcription levels of rmp genes and constructed a *rmpD*-deficient mutant to clarify whether the inhibition effect of IFA on HMV is associated with inhibition of capsule or rmp genes (please see the details in the response to next comment and details in the minor part). Additionally, considering that evidence regarding the capsule polysaccharide length is insufficient, we removed all the statements about this issue. Please see lines 151-153 and 175-181.

3, Nearly every experimental outcome is related to the reduced CPS/loss of HMV but the distinction, if any, between these two is not clear and I think is an important point to consider. Presumably this strain contains the rmpADC genes since it is HMV positive? Addition of a rmpD mutant could help elucidate whether the IFA-mediated virulence phenotypes are due to loss of HMV, reduced capsule, or potentially both (a rmpD mutant would be predicted to be HMV negative but have normal capsule production). This mutant would not need to be tested in all assays, but perhaps in some tissue culture assays and one of the in vivo models (mouse recommended, but I appreciate the time & expense may be prohibitive).

**Response:** We agree with and appreciate your insightful comments. As you noted, HMV requires both capsule synthesis and the small protein RmpD^1, 2^, it is subjective to overemphasize that IFA attenuates *K. pneumoniae* pathogenicity and the HMV phenotype through inhibiting capsule. Consistent with your speculation, the K7 strain used in the present study contains *rmp* genes (https://www.ncbi.nlm.nih.gov/nuccore/NZ_NKQH01000039.1). Given that *rmpD* has been widely recognized as an HMV regulator required for HMV but not capsule production, we constructed a *rmpD-*deletion mutant to precisely verify whether IFA-mediated virulence phenotypes are attributed to the reduction in capsule, the loss of HMV, or potentially both. The knockout method was included in the text. Please see lines 452-462 and 766-789.

Our new experiments demonstrated that the deletion of *rmpD* led to the total loss of HMV, as evident by the semiquantitative sedimentation assay and negative phenotype of “string test” shown below, and the above results are presented as S3 Fig A-C. Please see the legends in lines 801-824.

Next, we examined the effects of IFA on the capsule production, bacterial adhesion and phagocytosis, as well as the virulence of *ΔrmpD* K7. First, the results indicated that IFA still significantly reduced capsule production of *ΔrmpD* K7 under same culture condition as did wild-type K7, and the result are shown below and presented as S3 Fig D. Second, our new results confirmed that IFA also effectively increased the adhesion and phagocytosis of *ΔrmpD* K7 (the results are shown below and presented as S3 Fig E and F), indicating that IFA indeed hindered the immune evasion of hvKP through targeting capsule, rather than rmpD. Please see lines 244-250.

Last, mice infection experiments demonstrated that the deletion of *rmpD* resulted in a significant loss of virulence versus wild-type K7, as 1 × 10^7^ cfu (a 100% lethal dose used in wild-type K7) mutant bacterial didn’t cause death in mice (shown below). Surprisingly, 5 × 10^8^ cfu (50 folds of the inoculation dose used in wild-type K7) mutant bacterial didn’t cause death within 72 hours (shown below). Therefore, we performed bacterial burden assay by challenging 5 × 10^7^ cfu bacterial (20 folds of the inoculation dose used before). Our results demonstrated that the bacterial burdens of *ΔrmpD* K7 were much lower compared to wild-type K7 but significantly higher than *GT-1 K7,* which confirmed the distinct contribution of HMV and capsule and indispensable role of capsule in hvKP pathogenicity. Surprisingly, although IFA treatment reduced bacterial colonization of *ΔrmpD* K7 in mice lungs by about 6 folds versus vehicle group (*P*=0.0618), and by 4 folds in livers (*P*=0.0716), neither of them achieved statistical significance. By contrast, bacterial loading in spleens was significantly reduced by IFA (*P*=0.0211), indicating that the in vivo protection effect of IFA on hvKP infection is attributed to its inhibition on both capsule and hypercapsule-associated HMV. These results are shown below and presented as S3 Fig G-J. In addition, related results statements and discussion are included in the revised text. Please see lines 317-331 and 391-403.

Taken together, these supplementary material and results clearly elucidated that IFA impaired hypercapsule-associated mucoviscosity phenotypes and pathogenicity of hvKP via inhibiting capsule production, and the in vivo protection effect of IFA on hvKP infection is attributed to its inhibition on both capsule and hypercapsule-associated HMV, rather than rmpD. The results have been shown as S3 Fig and detailed results statements are also included as mentioned above.

4, Please carefully review literature and ensure the appropriate references are cited. I believe a few statements were mis-referenced and at least one typo was noted.

**Response:** Thank you again for your careful review. We have carefully checked the issue you raised and reviewed the mistakes in the citations and result statements. Please see the revised text.

5, This manuscript could benefit from some English language editing. There are a few non-sensical sentences and others with poorly-chosen or inappropriate descriptions that make it challenging to know what the authors are trying to communicate.

**Response:** Thanks for your constructive suggestion. We have addressed the awkward and confusing descriptions in the manuscript. To improve the English language, we polished the paper language at AJE again according to your comment, the certification is attached below.

6, I believe it is PLoS policy that all data need to be accessible? I think this would mean the RNA-seq data presented in Fig 3 should be publicly available, and available to the reviewers.

**Response:** We agree with and appreciate your constructive comment. Since the raw data of RNA seq are quite large, we did not upload these data. The RNA-seq data in the study have been deposited in the National Center for Biotechnology Information BioProject database and will be available in the Sequence Read Archive (SRA) under BioProject accession number PRJNA1108905 upon publication. The link below to the BioProject and associated SRA metadata is ready to copy and share with you to access the data: <https://dataview.ncbi.nlm.nih.gov/object/PRJNA1108905?reviewer=1dkapn599s26fql7renrj0ge08> Please see the data available statement in lines 640-644.

**Part III – Minor Issues**

**Reviewer #1**:

1, Please clarify the phrasing of “TLR4-govern bacterial clearance-promoting immune responses”, specifically stated in Lines 54-55 but also mentioned similarly throughout the manuscript. Do the authors mean to say “TLR4-independent clearance of bacteria”? The current phrasing is confusing.

**Response:** We express our gratitude again. According to your suggestion, we have removed experiments and results statements concerning TLR4-mediated immune responses. Please see the new Figure 5 and lines 231-252, 861-872.

2, The sentence in Lines 75-78 is not supported by the indicated reference. Please update to the correct citation.

**Response**: We apologize for this basic mistake. The correct reference has been cited in the revised manuscript. Please see line 78.

3. In the introduction, the authors emphasize the importance of CR-hvKp. Is the K7 strain used in this paper part a CR-hvKp strain? If so, this should be stated in the methods. If not, the introduction should be expanded to the importance of hvKp in general so as not to make the results seem relevant only to CR strains.

**Response**: We agree with and appreciate your comment. We have tested the MICs of common clinical antibiotics against K7 strain and the results are listed in the table below. Except for penicillin, lincomycin and erythromycin, K7 is susceptive to several other widely used antibiotics (with MICs ≤ 2 μg/ml), including imipenem. Therefore, the K7 strain used in our study is not a CR-hvKP. We have added this information in the methodology section, please see lines 444.

| The MIC values of different antibiotic against *K. pneumoniae* K7 | |
| --- | --- |
| Antibiotics | MIC value |
| Imipenem | 1 |
| Doxycycline | 1 |
| Gentamicin | 2 |
| Penicillin | 64 |
| Lincomycin | ＞1024 |
| Tetracycline | 2 |
| Erythromycin | 256 |

Additionally, we have reduced the descriptions of CR strains and emphasized the clinical importance of hvKP in the introduction. Please see lines 80-89.

4. It would be interesting to include background on the drug screen that led to the identification of IFA. Perhaps a Supplementary figure could detail the design and top hits. This could also be a good resource for noting additional compounds that influence capsule inhibition.

**Response**: We agree with and appreciate your comment. According to your suggestion, we have added the screening procedure and initial screen top hits as S1 Fig. Detailed information is also included in figure legend and the main body of revised manuscript. Please see the updated figures and lines 135, 758-760.

5. Figure 1 uses multiple methodologies to indicate that IFA represses capsule. Of these approaches, the staining in 1E is the most subtle. I would suggest removing this panel as the data is much clearer in 1C and 1F.

**Response**: We agree with and appreciate your constructive comment. As you mentioned, compared to the result from TEM (transmission electron microscopy) observation, the Anthony’s capsule staining is indeed subtle under current magnification condition. Therefore, we have removed the redundant data. Please see the updated Figure 1 and lines 135-143.

6. In Figure 2C, how were the centrifugation values assessed? In Line 178 the authors describe “approximately one-third”, but there is no quantitative measure in the figure. If the data is qualitative only, then the quantitative language should be removed.

**Response**: Thank you again for your careful review. As you indicated, the Fig 2C is just a qualitative data, we have removed this result according to the comment from Reviewer #3, since it did not add any new information for our conclusion. Please see the new Figure 2 and revised text.

7. In Figure 4, the immunofluorescence is quite difficult to see, especially for the red channels. Perhaps a software like ImageJ could be used to quantitatively measure these signals. The authors should certainly include a secondary-only antibody control to confirm that the straining in question is not non-specific binding of the secondary antibody.

**Response:** We agree with and appreciate your constructive comment. Firstly, we have added the quantitative immunofluorescence results as S4 Fig according to your suggestion, and the data is shown below. Please see lines 548-550 and 791-794.

Second, we performed complement binding experiment again to test whether the secondary antibody used in the study could non-specifically bind to bacteria surface. Given that the primary antibody targeting C3b/iC3b is a directly labeled antibody conjugated with allophycocyanin (APC), only the green immunofluorescence of Alexa Fluor 488 was checked.

As shown in the panel below, no visible green fluorescence was observed when the samples were directly incubated with Alexa Fluor 488-conjugated goat anti-mouse IgG after 3-hours serum exposure, whether for wild-type K7 or acapsular strain. Conversely, considerable green fluorescence was located on the surface of acapsular strain bacteria when the sample was first incubated with the primary antibody targeting C5b-9 and then incubated with the secondary the antibody (previous result), the results indicated that the staining of C5b-9 was not resulted from non-specific binding of secondary antibodies.

In fact, we included a serum-free control to eliminate the interference from the non-specific binding of antibodies, and the result demonstrated that there was no non-specific antibody binding in the experiment (shown in below), but we did not display this result since Figure 4 was already large enough, and the non-specific binding was internally excluded via flow cytometry-based detection. Additionally, the primary antibody targeting C3b/iC3b is a directly labeled antibody conjugated with allophycocyanin (APC), whereas the C5b-9 antibody requires a secondary antibody, displaying the second-only antibody control would be confusing. As a consequence, we have included only the serum-free control in the immunofluorescence microscopy analysis according to your comprehensive suggestion, and we hope that the results meet your requirements. Please see the new Fig 4 and lines 548-550.

8. It has been previously noted that acapsular strains have similar intracellular survival within macrophages compared to WT strains (PMID 26045209). The authors should note this in the discussion as it may influence their findings surrounding TLR4 pathways.

**Response:** We agree with and appreciate your comment. Although, we have deleted the results related to TLR4 pathway according to your suggestion, the important information and the reference you mentioned above were also added to our discussion. Please see lines 396-397.

9. The phrasing “IFA impeded the suppressive effect” in Lines 296-297 should be clarified as the double negative is confusing.

**Response:** Express our gratitude again. We have revised the title to “IFA impedes capsule-mediated immune evasion of *K. pneumoniae*”. Please see lines 231.

10. In Line 319, the word “reliable” is not quite correct. Galleria models are appropriate because they are accessible and feasible, but since these animals lack innate and adaptive responses of mammals, they are not necessarily used due their reliability.

**Response:** Thank you for your careful review. We have deleted this meaningless sentence. Please see lines 254-255.

11. The Galleria model should be explained for basic readout. For example, in Line 332 what does it mean for there to be an increase in melanization? How does this influence the readout of pathogenicity?

**Response:** We agree with and appreciate your comment. The release of lactate dehydrogenase as a marker of cell damage is referred as a critical predictor of virulence, and melanization is often defined as a marker of the insect innate immune system correlated with the pathogen’s ability to proliferate within *Galleria* and evade immunity, and thus is broadly correlated with poor survival^3^. We have explained these fundamental readouts in the revised text, please see lines 265-272.

12. In Line 338, the word “established” in incorrect. Murine pneumonia models have been extensively and widely used throughout the field to study K. pneumoniae lung infections.

**Response:** We agree with and appreciate your rigorous comment. We have replaced the word “established” with “used”. Please see lines 275-276.

13. In Figure 8F, each panel should be labeled. It is unclear which groups are being referred to.

**Response:** We apologize for this basic mistake. The panels have been correctly labeled according to your suggestion. Please see the new Fig 7F.

**Reviewer #2**:

1. Lines 45-46 are phrased awkwardly.

**Response:** Thanks for your careful review. We have revised the presentation according to your suggestion. Please see lines 45-46.

2. Lines 54, 253, - replace "-govern" with "-mediated."

**Response:** Thank you for your careful review. According to the suggestion from the editors and other reviewers, we have removed the experiments and results statements about TLR4-mediated immune responses as described in the response to major issues. Please see the new figures and text.

3. Lines 66, 401 - omit "great epidemic." Kp infection does not meet the classical definition of an epidemic.

**Response:** We agree with and appreciate your professional comment. We have revised inaccurate wording to “extensive spread”. Please see lines 66 and 339.

4. Lines 203-204 - these statements are not supported by image quantification or statistical analysis.

**Response:** We agree with and appreciate your comment. We have performed gray value analysis for Fig 1D using Image J and shown the statistical analysis as S2 Fig. Please see the new figures and lines 139, 483, 760-762.

5. Line 248 - "significantly" indicates image quantification or statistical analysis, but none is shown.

**Response:** We agree with and appreciate your comment. The image quantification has been performed using image J as you suggested, and the information has been detailed in in methodology section. Due to the large size of Fig 4, this result is shown as S4 Fig. Please see the new figures and lines 227 and 789-792.

6. Line 255 - this sentence needs revision.

**Response**: Addressed as described in the above response to Q2, we have removed those redundant results. Please see the revised text.

7. Line 263 - "MPM" is not defined.

**Response**: We express our gratitude again for your careful review. Although the sentences in line 263 have been deleted, we checked and revised the issue you raised throughout the manuscript, please see lines 235, 560, 797, 869.

8. Line 268 - typo in TLR.

**Response**: Addressed as described in the above response to Q2, we have removed the results about TLR4-mediated response. Please see the revised text.

9. Lines 290-295 - I do not think that the authors have provided sufficient evidence to support their conclusion. It may be that the ratio of different PAMPs (LPS, capsule, etc.) modulate the interaction with TLR4, rather than it being specific to capsule recognition of TLR4.

**Response**: We agree with and appreciate your comment. As described in the response to Q2, we have removed the redundant result concerning TLR4-mediated responses. Please see the revised text.

10. Line 391 - most tlr4-/- mice appear to be dead at 32 hours in Figure 10A.

**Response**: Thank you again for your careful review. Although we have deleted this part, we would like to explain to you that in the experiment, mice were challenged with a sublethal dose of 2 × 10^6^ cfu *K. pneumoniae* K7 and anesthetized at 32 hours post infection when they were still alive.

11. Line 391/3 - IFA enhances, rather than inhibits, expression of these genes.

**Response:** We apologize for the basic mistake. As described in the above, we have removed the redundant result about TLR4-mediated responses. Please see the revised text.

12. Lines 391-392 - there does appear to be IFA-mediated enhancement of Mx1 expression in Figure 10D. Please clarify.

**Response**: As described in the response to Q2, we have removed the redundant result. Please see the revised Figure 5 and lines 231-252, 861-872.

13. Lines 485-486 - which drugs are the authors referring to?

**Response**: We agree with and appreciate your constructive suggestion. We have listed some host-directed drugs regards bacterial replication and activating immune response in the discussion, for example, the autophagy-inducing drug bromhexine hydrochloride^4^**,** and the bioactive lipid phosphatidylinositol 5-phoshate (PI5P)^5^ . Please see lines 418-419.

14. Line 510 - please provide methodological details for the capsule mutant or the relevant citation where it was created.

**Response**: We agree with and appreciate your constructive suggestion. The knockout method is elaborated in detail in the methodology section. Please see lines 447-449

15. Lines 551-552 - where are MIC values reported?

**Response:** Thanks for your careful review again. We have added this missing result to the manuscript (the MIC of IFA against K7 *K. pneumoniae* exceeded 1024 μg/ml). Please see lines 135-137.

16. All figures, please show exact p-values.

**Response:** Express our appreciate again. We have added the P-value to all the statistical panels. Please see the revised figures.

17. For figure 1C, 1H-I, 2A-B, 2E, 3F-G, 4F-H, 5A-J, 6, 7C-D, 8G-J, 9 A-E, 9F, 9G, and 10B-K, please show individual datapoints.

**Response**: We agree with and appreciate your constructive suggestion. The individual data points are included in all the figures you mentioned. Please see the new figures.

18. Figure 2A - the x-axis label is cut off.

**Response**: Thank you for your patient review. We have corrected this mistake. Please see the new Figure 2.

19. Figure 2B - the y-axis is mislabelled.

**Response**: We apologize for this basic mistake again. We have revised the wrong y-axis label to “Hypermucoidy (supernatant OD_600_/input OD_600_)”. Please see the new Figure.

20. Figure 2C - the statements in the text require quantification to be supported.

**Response**: Thanks again for your careful review. According to previous study^6^, Percoll gradients centrifugation of bacterial shown in Figure 2C is defined as a qualitative data rather than quantitative data. Given that the result seems somewhat redundant to the data shown in Figure 2A and 2B (according to the suggestion of Reviewer #3), we remove this panel from Figure 2.

21. Figure 3C lacks clear labeling. Presumably "C" and "T" refer to control and treatment?

**Response:** We agree with and appreciate your careful review. As you guessed, C refers to the control group, and T refers to the IFA-treated group. The correct labels have been included in Figure 3C according to your recommendation. Please see the new figure.

22. Figure 4A-B - the axis labels are condensed to the axis.

**Response**: Thank you for your careful review, we have revised these two panels, please see the new figure.

23. Figure 5K-M - it is unclear which groups are being compared.

**Response**: Thanks again for your careful review. We have removed the 8 µg/ml group according to the suggestion from Reviewer #3 and added clear comparisons between different groups. Please see the new Fig 5.

24. Figure 8F - please label each histology image.

**Response**: Apologize for the basic mistake again. We labeled each histology image in Figure 7F. Please see the new Figure.

25. Figure 9F-K - please clarify the y-axis. Do the authors mean % of total cells for each marker set?

**Response**: Thanks for your careful review. The y-axis in Figure 9F-K means the percentage of gated 30000 cells from individual mice BALF for each marker set. The details are clarified in the figure legends. Please see lines 615-616, 918-919.

26. All figure legends - the figure legends indicate a comparison to vehicle control, but there are comparison bars on many figure panels. Please clarify.

**Response**: Thank you again for your careful review. We have checked the issue you raised throughout the text and carefully revised inaccurate legends and comparisons. Please see the new figures and lines 814-816, 827-829, 838-840, 870-872, 886-887, 906-907 and 920-921.

27. Table 1 does not need to be in the main body.

**Response:** We agree with and appreciate your constructive suggestion again, Table 1 has been moved to Supplementary information.

**Reviewer #3**:

1. Fig 1C, this is a relatively small decrease in UA. What amount of UA is detected in a capsule mutant? Knowing that baseline UA level would help interpret the significance of the decrease from IFA treatment.

**Response:** We agree with and appreciate your scientific question. We’ve repeated UA assay which included the acapsular mutant. As shown in the left panel below, although IFA significantly reduced the total UA level compared to vehicle group, but it failed to reduce UA level to that of acapsular mutant at 32 μg/ml, which is approximately in the middle between the wild-type and acapsular mutant. It is worth mentioning that IFA-treated wild-type K7 exhibited similar capsule thickness versus acapsular strain in overall under TEM observation (right panel), thus we speculate that the significant difference in total UA level compared to acapsular baseline might be attributed to the accumulation of unattached capsular polysaccharides during culturing. Given that the inclusion of acapsular mutant might influence the logical continuity of Fig 1, we finally didn’t show this group in Fig 1C (new Fig 1B). We would greatly appreciate your understanding.

2. Fig 1D, I agree with the authors that there is a decrease in the amount of high molecular weight capsule in this gel. However, it does not show the shift to lower molecular weight that occurs with loss of HMV as reported by Walker, et al. 2023 and Khadka, et al. 2023. With out that observation, it cannot necessarily be concluded that IFA reduces chain length. (example of an overstated conclusion mentioned above)

Response: Thank you for your careful review. We have deleted those non-sensical sentences and overstated conclusion. Please see lines 137-139.

3.Fig. 1E, these images are not very effective. It is almost impossible to see the bacteria unless zoomed way in and even harder to see the CPS staining. I do not think this data adds to the already convincing data that IFA reduces CPS.

**Response:** We agree with and appreciate your constructive question. The Anthony’s staining result has been removed from Fig 1 according to your suggestion. Please see the new Fig 1.

4.Fig. 1G, it is unclear how the CPS thickness would be measured between 100-200 nm based on the image provided, where it looks to be maybe 50 nm. Can the authors clarify this discrepancy? I recognize this is a representative image, but if the data are going to be quantified and graphed, the representative image should match the data in the graph.

**Response:** We apologize for the rough measurement. The CPS thickness was incorrectly measured using the way shown below. According to your suggestion, we repeated the quantification by measuring the capsule thickness from the bacterial membrane to the top of the CPS. Please see the revised Figure 1F.

5.Fig 2A, what is the rationale for two different centrifugal forces? No mention was made regarding these two forces, and I personally think one is sufficient to illustrate the HMV results. However, if both are to be shown, the Y-axes should be the same on these two graphs to better illustrate the differences.

**Response:** We agree with and appreciate your comment. In accordance with a previous study, 1000 × g was applied as the centrifugal force to define decreased hypermucoidy relative to that of the wild-type strain, while the higher force exceeds 2500 × g was aimed to identify increased hypermucoidy^6^. Thus, we deleted the result of 5000 × g as you recommended. Please see the new Figure 2.

6． Fig 2B, Y-axis label is incorrect

**Response**: We apologize for this basic mistake. We have replaced the wrong label with “Hypermucoidy (supernatant OD_600_/input OD_600_)”. Please see the new Figure 2.

7． Fig 2C, it is not clear what the authors are attempting to demonstrate with this experiment. The statement in the results (lines 181-182) does not articulate a conclusion. It also seems somewhat redundant to the data shown in 2A,B. Recommend removing this unless there is new information that can be obtained from this.

**Response:** We agree with and appreciate your constructive suggestion. Fig 2C and related-statement have been removed according to your comment. Please see the new Fig 2 and revised text.

8． Fig 2E, is redundant to 2A and adds no new information. What is the purpose of fold change in string test length? FC relative to what? This assay is highly subjective and should not be quantified. The sedimentation assay (as in 2A,B) is more quantifiable. Given the overwhelming amount of data in this paper, I recommend removing this (or just stating in Results).

**Response:** We agree with and appreciate your constructive comment. According to your suggestion, we have deleted the redundant data in Fig 2E. Please see the new Figure 2.

9. Fig. 2F, it appears that for both K7 and ∆GT, the colonies on plates with IFA are smaller, suggesting a growth defect. Is this the same medium as used in Fig. 1B? This potential discrepancy warrants explaining.

**Response**: Thanks for your patient review. As you mentioned, the medium used in Fig 1B was Luria-Bertani broth, which lacked only 1% agar compared with the medium used in Fig. 2F. To explore whether the deletion of *GT-1* caused growth defect, we measured the growth curves of wild-type K7 strain and acapsular *∆GT-1* K7 strain, respectively. As shown below, their growth curves in LB medium are almost identical, indicating that GT-1 is not required for bacterial growth. In addition, neither of them was affected by IFA.

We apologize for the confusion about colony morphology. The images shown in Fig. 2F were taken from bacterial plating on LB agar plates, and original data are shown in below. We recognize that the minor difference in *∆GT-1* K7 strain should be resulted from the discrepancies in focal length, as shown in the original image below, the colony sizes of wild-type K7 on IFA containing LB agar plate were much smaller overall compared to vehicle control, whether for 8 ug/ml and 32 ug/ml, while such significant reduction was not observed in *∆GT-1* strain.

Together, these results confirmed that IFA-caused reduction in K7 colony sizes was attributed to CPS reduction, rather than bacterial growth.

10. Lines 201-203 the wrong reference is given, and I believe the authors are mis-stating some findings.

**Response**: Thank you for your patient review. We have removed those non-sensical sentences. Please see the revised text.

11. Ref 22 has author list/title that do not go together.

**Response**: Express our gratitude again. We have corrected the reference in the revised text. Please see line 193.

12. Fig 3 in general I found to be a bit of a distraction. Beyond demonstrating that the correlation between ATP levels and HMV status occurs in this strain (which is interesting!), the rest of this data was not particularly informative without further follow-up. At a minimum, I think it worth commenting whether expression of capsule synthesis genes or the rmp genes were impacted by IFA.

**Response**: We agree with and appreciate your constructive comment. As you suggested, the HPLC analysis of capsular polysaccharide in Fig 3 did not provide very helpful evidence for how IFA reduces CPS thickness, thus the panel Fig 3A has been deleted from the new Figure. Given that the discovery of a potential correlation between ATP levels and HMV/CPS thickness was based on the RNA-seq analysis, we retained some transcriptome results in the new figure, except for KEGG pathway analysis in Fig 3E. In addition, a large body of published work has shown that the NAD+/NADH redox balance regulates metabolic status and energy production^7-9^, and that the ratio of NAD+/NADH is positively correlated with ATP production, therefore we retained the experiment.

In particular, we detected the expression of CPS synthesis genes and rmp genes according to your scientific suggestion. As shown below, IFA treatment resulted in significant decrease in the transcriptional levels of capsule synthesis genes *gnd, wcaJ, wbaP, wzx, wza, wab, rcsB* and *rfaH* versus vehicle group, while *rmpA/C/D* were not affected. The result has been included in new Fig 3. Please see the new figure and revised text, lines 198-204.

13. The title to Fig 3 is misleading. There is no data in this figure that relate to chain length, only the global impact of IFA on Klebsiella transcription.

**Response**: We agree with and appreciate your careful suggestion. We’ve replaced the title with “IFA reduced the capsular biosynthesis through altering bacterial metabolism” in both the main body of text and figure legend. Please see lines 182 and 831.

14. Fig 4A, B, all other figures so far use 32 µg/ml. Why 64 here?

**Response:** Thank you, we fully understand your doubts about this discrepancy.

Although we have spectroscopically detected the growth curves of *K. pneumoniae* strain K7 with the treatment of IFA ranging from 4-64 μg/ml, we believe that it is necessary to further confirm that IFA inhibits serum survival of *K. pneumoniae* through targeting capsule, but not its antibacterial activity, where the number of bacterial was determined by microbiological plating. Therefore, we included 64 µg/ml in Fig 4A and 4B. If necessary, we could remove the 64 µg/ml group.

15. Fig 4C, D, what is the purpose of the lower set of images (presumably bright field, but this is not mentioned in the legend)? Nothing can be seen in the panel C T1 images.

**Response:** Thanks again for your careful review. The lower set of images in Fig 4C, D were merged by the fluorescence field and bright field, which could help determine complement binding level on the bacterial surface and avoid unspecific binding by co-localizing the bacterial morphology and fluorescence spot. Detailed information has been added to the legend, please see lines 852-853.

In addition, the wild-type K7 strain indeed exhibited almost no fluorescence staining during the first hour (T1) owing to capsule-mediated resistance, regardless of treatment, while a small amount of red/green fluorescence could be observed in the acapsular strain panels. After a 3-hour incubation, stark decreases were observed in IFA-treated K7 strain and acapsular strains compared with wild-type strain. We have analyzed the fluorescence intensity of T1 and T3 using Image J software. Please see Fig 4, S4 Fig and lines 223-225.

16. Fig 4 title is mis-leading. Capsule is not eliminated by IFA

**Response**: We agree with and appreciate your rigorous comment. We’ve revised the title to “IFA sensitized hypervirulent *K. pneumoniae* to complement-mediated killing by suppressing capsule”. Please see line 842.

17. Fig 5A-J, please provide the gene name in a prominent place (such as with panel letter, or upper left corner of graph). These fonts are so small they can only be read when zoomed in.

**Response**: Thanks again for your careful comment. Addressed as described in the above response to Summary section, we have removed the experiments on TLR4-mediated immune responses according to the suggestion from you and editors, which made the manuscript more readable and streamlined. Please see the new Fig 5.

18. Fig 5K-M, please keep Y-axes the same in all graphs for better comparison. I would also remove the 8µg/ml treatment as it does not add anything.

**Response**: We agree with and appreciate your constructive comment. According to your suggestion, we kept same Y-axes in Fig 5K-M, and removed the 8 µg/ml treatment group. Please see the new Fig 5.

19. Fig 7C are K7 and K7+IFA mis-labeled?

Would be helpful if the color scheme in Fig 7 matched Fig 8&9

**Response**: Sorry for the basic mistake again. The K7 and K7+IFA groups were indeed incorrectly labeled before, and the panel has been revised with correct label. Additionally, we agree with and appreciate your careful comment on the color scheme. According to your suggestion, the color scheme has been changed to that of Fig 8&9. Please see the new Fig 6.

20. Fig 8F, please label each part with the strain used. There are 4 strains/conditions but 5 images. Presumably one is uninfected?

**Response**: Thanks for your careful review. As you speculated, the first panel is an uninfected control and we have added the missing label for each panel. Please see the new Fig 7.

21. Fig 9F-K, please label the individual flow cytometry panels with the strain/condition.

**Response**: We express our gratitude again. The panels have been correctly labeled. Please see the new Fig 8.

**References:**

1.	Ovchinnikova, O. G.; Treat, L. P.; Teelucksingh, T.; Clarke, B. R.; Miner, T. A.; Whitfield, C.; Walker, K. A.; Miller, V. L., Hypermucoviscosity Regulator RmpD Interacts with Wzc and Controls Capsular Polysaccharide Chain Length. *Mbio* **2023,** *14* (3).

2.	Walker, K. A.; Treat, L. P.; Sepúlveda, V. E.; Miller, V. L., The Small Protein RmpD Drives Hypermucoviscosity in. *Mbio* **2020,** *11* (5).

3.	Wand, M. E.; McCowen, J. W. I.; Nugent, P. G.; Sutton, J. M., Complex interactions of

with the host immune system in a infection model. *J Med Microbiol* **2013,** *62*, 1790-1798.

4.	Choi, S. W.; Gu, Y. X.; Peters, R. S.; Salgame, P.; Ellner, J. J.; Timmins, G. S.; Deretic, V., Ambroxol Induces Autophagy and Potentiates Rifampin Antimycobacterial Activity. *Antimicrob Agents Ch* **2018,** *62* (9).

5.	Poerio, N.; Olimpieri, T.; De Angelis, L. H.; De Santis, F.; Thaller, M. C.; D'Andrea, M. M.; Fraziano, M., Fighting MDR- Infections by a Combined Host- and Pathogen-Directed Therapeutic Approach. *Front Immunol* **2022,** *13*.

6.	Dorman, M. J.; Feltwell, T.; Goulding, D. A.; Parkhill, J.; Short, F. L., The Capsule Regulatory Network of Defined by density-TraDISort. *Mbio* **2018,** *9* (6).

7.	Im, H.; Pearson, M. L.; Martinez, E.; Cichos, K. H.; Song, X. H.; Kruckow, K. L.; Andrews, R. M.; Ghanem, E. S.; Orihuela, C. J., Targeting NAD plus regeneration enhances antibiotic susceptibility of during invasive disease. *Plos Biol* **2023,** *21* (3).

8.	Rodionova, I. A.; Schuster, B. M.; Guinn, K. M.; Sorci, L.; Scott, D. A.; Li, X. Q.; Kheterpal, I.; Shoen, C.; Cynamon, M.; Locher, C.; Rubin, E. J.; Osterman, A. L., Metabolic and Bactericidal Effects of Targeted Suppression of NadD and NadE Enzymes in Mycobacteria. *Mbio* **2014,** *5* (1).

9.	Rivera-Lugo, R.; Deng, D. V.; Anaya-Sanchez, A.; Tejedor-Sanz, S.; Tang, E. G. E.; Ruiz, V. M. R.; Smith, H. B.; Titov, D. V.; Sauer, J. D.; Skaar, E. P.; Ajo-Franklin, C. M.; Portnoy, D. A.; Light, S. H., requires cellular respiration for NAD regeneration and pathogenesis. *Elife* **2022,** *11*.
