# Supplementary material for: Isoferulic acid facilitates effective clearance of hypervirulent Klebsiella pneumoniae through targeting capsule
Source: PLoS Pathog. 2025 Jan 6;21(1):e1012787. doi: 10.1371/journal.ppat.1012787 (PMC11737856; doi:10.1371/journal.ppat.1012787)
Supplement: Attachment — Submitted filename: Point to point response.docx [file ppat.1012787.s007.docx]

**Response to the editors**

Dear Dr. Michael Malim, Dr. D. Scott Samuels and Dr. M Ammar Zafar, thank you very much for all your input into our revised manuscript (PPATHOGENS-D-24-01091_R1). We have addressed your concern about the vehicle control and added detailed description for vehicle-only solvent control throughout the manuscript. We sincerely appreciate the comments that you and the reviewers provided, and the manuscript has been revised according to the reviewer’s comments. We hope that the revised manuscript has met the editorial requirements you and the reviewers described and fully met the journal’s publication criteria. Your attention to our revised manuscript is sincerely appreciated. The following are our responses to the reviewer’s comments.

**Response to Reviewers**

We would like to express our sincere gratitude for all the positive and professional comments the reviewers provided.

Part I – Summary

Please use this section to discuss strengths/weaknesses of study, novelty/significance, general execution and scholarship.

Reviewer #1: In this revised manuscript by Wang, et al., the authors have substantially improved their original article and clearly modified the manuscript to address many reviewer concerns. The study is certainly more streamlined in the revision by removing redundant experiments and tangential data.

While this manuscript has been substantially improved, I remain skeptical about the off-target effects of the vehicle control. Further, I remain unconvinced that the authors have uncovered novel biology in Figures 4-8 as these experiments largely confirm previous findings reported by other groups when studying the effects of acapsular strains on host response.

Response: We sincerely appreciate your scientific and professional comment.

(1) In our study, IFA was dissolved in DMSO for *in vitro* experiments, and was dissolved in a 10% DMSO containing vehicle solvent (consist of 10% DMSO, 45% stroke-physiological saline solution, 40% PEG400 and 5% Tween-80, which referred to as IFA vehicle in your comments) for most *in vivo* experiments except for the LDH assay. In mouse infection experiments, 30 μl bacterial suspension in PBS containing indicated amounts of bacteria was intranasally inoculated to each mouse for infection, and the mice in vehicle-only control group were also treated with equal volume of PBS via intranasal route. Similarly, *Galleria mellonella* were treated in the same way to set infection and control groups.

As you mentioned, the vehicle control might have off-target effects on host, thus, the animals in vehicle-only control group (no infection) and vehicle control (with infection) received the same volume of solvent that dissolved IFA throughout the study. Of note, the content of DMSO in solvent vehicle was reduced from 10% to 1% (1% DMSO, 54% stroke-physiological saline solution, 40% PEG400 and 5% Tween-80) to exclude the potential toxic effect of DMSO on Galleria mellonella in LDH assay, and IFA was also dissolved in the same 1% DMSO containing vehicle to make a 1.5 mg/ml solution in this experiment. Eventually, 10 μl of vehicle or IFA were injected to the larvae (50 mg/kg). Therefore, we always ensured the consistency of the vehicle throughout each *in vivo* and *in vitro* experiment. Herin, we recognize that more detailed and clearer statements are indeed required for vehicle control throughout the manuscript. Moreover, to avoid any confusion, we have replaced “vehicle” with “DMSO” in the *in vitro* experiments. Please see lines 437-440, 602-603, 789-791, 840-849, 884-886, 896-898, 905-907 and 928-939.

(2) In addition, the study focus mainly on defining IFA as a broad-spectrum inhibitor of *K.P* capsule, exploring its potential underlying mechanism and subsequently investigating whether this chemical inhibitor could combat hvKP infection through attenuating capsule-mediated immune evasion, including resistance to serum killing, epithelial adhesion and phagocytosis of macrophages. Although some experiment in Figure 4, Figure 5 and Figure S2 were repetitive with previous studies, these results further confirmed the specific inhibitory effect of IFA on capsule synthesis and capsule-associated HMV, both of which are critical pathogenicity phenotype of HvKP. Building upon the above result, we further confirmed its *in vivo* protection effect and the results revealed that IFA significantly prevented hvKP infection through targeting capsule, which was mainly associated with effective immune clearance as mentioned above. Taken together, the results in Figures 4-8 further confirmed the specific inhibitory effect of IFA on capsule production and capsule-associated HMV, as evident by attenuated complement resistance and immune evasion (Figure 4-5 and Figure S2), and at the same time, the results in Figure 6-8 provided strong evidence that IFA could prevent hvKP infection as a broad-spectrum capsule inhibitor, which would benefit the development of capsule-targeted alternative therapeutic strategies aimed to curb hvKP infections.

According to your constructive comments, we have supplemented more discussion about drug screen and underlying mechanisms so as to provide some valuable insights for discovering more effective capsule and molecular mechanism research in the future. Please see lines 373-378.

Reviewer #2: This manuscript is a resubmission by Wang, Yang et al. reports the findings of a study exploring the effects of isoferulic acid (IFA) on Klebsiella pneumoniae (Kp) capsule expression and resulting phenotypes. The authors have addressed many of the reviewer requests, and the data demonstrate a high degree of rigor. Unfortunately, in the the revision process, the most novel data (autophagy and TLR4) was removed. By removing these data, this study shows that IFA represses capsule, which in turn, reduces virulence. While these data are highly convincing, the relationship between capsule, mucoviscosity, and virulence has been very well documented. Thus, in its current form this manuscript suffers from a lack impact.

Response: Thank you very much for your positive and constructive comments on the revised manuscript. In the first round of revision, the editor, Reviewer#1 and Reviewer#3 proposed that the study should focus mainly on defining IFA as a broad-spectrum capsule inhibitor, rather than exploring how IFA affected the interaction between *K. pneumoniae* capsule and host immune response, including capsule-specific engagement of TLR4 pathways and autophagy, because they believed that those results make the story not that readable and streamlined. Even more, the editors pointed out that the manuscript was weighed down by the experiments on the mechanism of innate immune responses. Whereafter, we removed the results associated with TLR4 and autophagy according to their suggestion. After a discussion with the editors, we kept the main body of R1 manuscript. We greatly appreciate your understanding of this discrepancy. Please refer to the detailed point-to-point reply below. We hope that this revised manuscript meets the requirements you described.

Reviewer #3: The revised manuscript by Wang, et al. (PPATHOGENS-D-24-01091_R1) is much improved. The removal of several redundant experiments has made this a more readable paper. The conclusions are more appropriate for the data presented, and the inclusion of the data with the rmpD mutant (Fig S3) and the transcript data (Fig 3F) clarify that IFA more specifically impacts capsule rather than HMV. I think most of the reviewer comments have been sufficiently addressed and I have just a few remaining questions and comments.

Response: Thank you very much for your positive comments on our revised manuscript and all your input into the revision process. Please refer to the detailed point-to-point reply below. We hope that this revised manuscript meets the requirements you described.

**Part II – Major Issues**

**Reviewer #1**:

1. In the response to reviewers, the authors state that the IFA vehicle is 10% DMSO, but that the experiments were carried out with vehicle "reduced DMSO to 1% and the PBS control was also treated with IFA vehicle". The vehicle control should be exactly what the vehicle is delivered in for the experimental condition. It is not appropriate to administer IFA with 10% DMSO but use 1% DMSO as the vehicle control. This is a log less of DMSO, a chemical that can influence the host. The authors should add the corrected DMSO control and clearly state that this control is used in each panel where the comparison is made.

Response: Thanks again for your rigorous and patient review. Addressed as described in the above response to Summary, we kept using the same vehicle solvent for vehicle-only control (no infection), vehicle control (with infection) and IFA treatment in each *in vivo* experiment throughout the study. Of note, in LDH assay, the larvae in vehicle control group were also treated with the 1% DMSO containing vehicle, and IFA was also dissolved in the vehicle containing 1% DMSO rather than 10% DMSO. According to your professional recommendation, we have replaced the “vehicle control” with “DMSO control” in all *in vitro* experiments and clearly stated the specific vehicle control used in each panel. Please see lines 437-440, 602-603, 789-791, 840-849, 884-886, 896-898, 905-907 and 928-939.

2. The majority of the experiments in this manuscript remain detailing the host responses to acapsular Kp and not in understanding specifically how IFA is decreasing capsule. I wish that the drug screen identifying IFA was in larger focus and that more space was dedicated to understanding properties of drugs that inhibit capsule synthesis and not on reconfirming previous findings.

Response: Thank you for your constructive comments. The study focus mainly on defining IFA as a broad-spectrum inhibitor of K.P capsule, exploring its potential underlying mechanism and subsequently investigating whether this chemical inhibitor could combat hvKP infection through attenuating capsule-mediated immune evasion, including resistance to serum killing, epithelial adhesion and phagocytosis of macrophages. Although some experiment in Figure 4, Figure 5 and Figure S2 were repetitive with previous studies, these results further confirmed the specific inhibitory effect of IFA on capsule synthesis and capsule-associated HMV, both of which are critical pathogenicity phenotype of HvKP. Building on the above result, we further confirmed its *in vivo* protection effect and the results revealed that IFA significantly prevented hvKP infection through targeting capsule (Figure 6-8), which was mainly associated with effective immune clearance that were validated above. In addition, we have supplemented more discussion about drug screen and underlying mechanisms so as to provide some valuable insights for discovering more effective capsule and molecular mechanism research in the future. Please see lines 373-378.

**Reviewer #3:**

1. Fig 1D is redundant, not terribly very convincing, and the quantification data are difficult to fully evaluate without a normalization step. Presumably equal ODs were loaded, but there is insufficient information about this. M&M state “equal amounts” but is this equal OD or equal culture volume?? If the authors wish to keep this in, I would remove the quantification. The uronic acid assay is sufficient to demonstrate a reduction in CPS.

Response: Thank you for your constructive comments. The statement “equal amounts” in Fig 1D means both equal OD and equal culture volume, in this way, we ensured the loading samples were extracted from equal amount of bacterial. Considering that the alcian blue staining of CPS in SDS-PAGE is a widely used method for analyzing the amount of capsule production and providing visualization result, we choose to remain this panel in Figure 1, but we removed the quantification result according to your suggestion. Please see the revised result and line 139.

2. Lines 160-161, the study referenced (Mike et al. 2021, ref 21) does not conclude that excessive CPS is required for HMV, just that some threshold level of CPS is required. This distinction was first reported by Walker, et al. 2020 (ref 36). It is true that some low CPS-producing mutants also lose the HMV phenotype, but it is important to distinguish that excess CPS and HMV can be distinct phenotypes. There may be some strains where it is hard to distinguish reduced CPS from HMV, and it is likely that the decrease in CPS caused by IFA in strain K7 drops below the threshold needed for the strain to become HMV positive. I would encourage cautious wording so as to not overgeneralize the findings.

Response: Thank again for your careful and insightful comments on this issue. We have cited correct references as you recommended and revised the statements that are not that stringent enough. Pease see lines 159-163.

3. RcsB and RfaH are major positive regulators of cps gene expression. That transcripts for both of these genes are significantly reduced suggests that IFA may be acting at a level upstream of cps gene expression. This information should be addressed in the Discussion.

Response: Express our gratitude again for your constructive comments. We have added this valuable information in the discussion as you suggested. Please see lines 390-392.

4. Fig 5A and S3E, adhesion data I think is more typically presented as a percent of the inoculum. If the inocula values are available, I recommend reanalyzing the data. It is curious to me that in 5A the deltaGT mutant has much lower adherence than K7+IFA and I wonder if this is reflective of differences in the inoculum?

Response: Thank you for your constructive comment, we sincerely appreciate your insightful evaluation on this result. Fortunately, we also determined the inoculum value at initial. As you speculated, this confusing observation was indeed resulted from the difference in inoculum (although we adjusted all the bacterial suspensions to OD _600_ = 0.1 and added equal volume of bacterial to each well). According to your constructive suggestion, we presented the results as a percent of the inoculum and yield a more reasonable result. The results are shown below. Please see the new Figure 5A, Figure S2E, and lines 569, 783-784, and 873-874.

 **Figure 5A	Figure S3E**

**Part III – Minor Issues**

**Reviewer #2:**

1. Please indicate which datapoint in S1B is IFA.

Response: Thank you for your constructive comment. We have indicated the datapoint of IFA in Fig S1B, and the new figure is shown below. Please see the revised figure.

2. Line 199 "gud" is a typo.

Response: Apologize for this basic mistake. We have corrected the typo. Please see the line 201.

3. Line 282 "huge" is not a common scientific term.

Response: We agree with and appreciate your professional comment. We have replaced the incorrect word “huge” with “high”. Please see the line 285.

Reviewer #3:

1. Line 97, I’m not sure this is the appropriate reference for the statement made. I would guess the information might be included in that paper, but a more appropriate reference would be one of the more recent reviews on CPS synthesis and export.

Response: We agree with and appreciate your professional comment. We have checked the latest research progress and reviews in this field once again, and confirmed that although many new capsule regulators have been identified in the past few years(1), the Wzx/Wzy-dependent pathway encoded by *cps* locus similarly to “group 1 capsule” in *Escherichia coli* is an well recognized capsule synthesis mechanism for *K. pneumoniae* (2, 3). It is worth noting that some gram-negative bacterial, such as *E. coli*, *Neisseria meningitidis* and *Haemophilus influenzae*, also use the ABC transporter-dependent pathway to synthesize capsule polysaccharide (4). Therefore, with continuous evolution, new capsule synthesis pathways may also be discovered in *Klebsiella pneumoniae* in some day. To avoid overgeneralization, we have revised the statement according to your comments. Please see lines 95-98.

2. Line 183, this section on transcriptomic changes due to IFA does not really address mechanism, as stated. It certainly provides insight, and narrows the target to cps gene expression and expression of cps regulators, but this is not “mechanism”. Please edit accordingly.

Response: Thanks again for your professional comment. We have revised these inaccurate statements according to your suggestion. Please see the line 185.

3. Line 215, the statement “while the amount of wild-type K7 began to decrease until 120 min” is somewhat confusing considering it decreases further at each timepoint, including 180 min. Please edit for clarity and accuracy.

Response: We apologize for the confusing description. We initially want to emphasize that the serum killing of wild-type K7 was obviously delayed compared to the capsule-deficient mutant and a sharp decrease was observed until 120 min, while the stark decrease occurred with 60 min in capsule-deficient mutant. We have revised this inaccurate statement according to your suggestion. Please see lines 217-218.

4. Fig 5, why are the MOI different for epithelial cells vs macrophages? I realize this will not alter interpretation, but the rationale should be stated.

Response: Thank you for your careful review. The MOI for adhesion and phagocytosis in our study referred to a previous study (5), and we speculated that the reason why a lower MOI was chosen for phagocytosis assay in many studies might be attributed to the active phagocytic function of macrophages and the limit of macrophage phagocytic ability. According to your patient comment, we have cited the reference in the method. Please see lines 562-563.

5. Please indicate the limit of detection and define the black lines (means, medians?) for the bacterial burden data in Galleria and mice (figs S3, 6, 7).

Response: We agree with and appreciate your constructive comment. We have indicated the limit of detection for bacterial burden in Figure S3, 6 and 7 according to your professional comment. Of note, when calculating the detection limit, we realized that the bacterial count in Figures 6B and 7B, 7D and 7E was underestimated by 100 times, because only 10 μl dilution was plated on LB agar plate for CFU enumeration, but we omit to calculate back to 1 ml for final bacterial density value. Additionally, we recognized that the bacterial burden of *△GT-1* K7, *△GT-1* K7 + IFA and K7 + IFA groups are below detection limit, because most of their CFU enumeration were 0 under the same experimental condition, to make the log10 data have mathematical meaning, we initially normalized those “log_10_^0^” values to 1 or 0. Now, we will normalize these ND (not detected) data to the detection limit and perform data analysis again based on the updated data. We need to remind here that the correcting will not alter our conclusion. We would greatly appreciate your understanding.

In addition, the back lines in new Figure S2, 6 and 7 presents the means ± SEMs, and we have added the detailed information throughout the manuscript as you suggested. Please see the new figures and lines 797-798, 891-892 and 919.

A few typos were noted:

(1) Line 169, reference to colonies on LB agar, but figure shows blood agar.

Response: Express our sincere gratitude again for your careful review. We have carefully checked and revised the description. Please see the. Lines 170-172.

(2) Line 199, replace gud with gnd

Response: Apologize for this basic mistake. We have corrected the spelling. Please see lines 201.

(3) Line 249, something is not correct here. Perhaps the use of “liberating” is not appropriate?

Response: We agree with and appreciate your scientific comment. We have replaced the inappropriate word “liberating” with “enhancement”. Please see line 252.

(4) Line 282, 321, 327, others?, folds should be fold (not plural).

Response: Thank you very much for your careful review. We have corrected this typo throughout the manuscript. Please see lines 320-330.

**References:**

1.	Dorman MJ, Feltwell T, Goulding DA, Parkhill J, Short FL. The Capsule Regulatory Network of lebsiella pneumoniae Defined by density-TraDISort. Mbio. 2018;9(6).

2.	Arato V, Raso MM, Gasperini G, Scorza FB, Micoli F. Prophylaxis and Treatment against Klebsiella pneumoniae: Current Insights on This Emerging Anti-Microbial Resistant Global Threat. Int J Mol Sci. 2021;22(8).

3.	Caffalette CA, Kuklewicz J, Spellmon N, Zimmer J. Biosynthesis and Export of Bacterial Glycolipids. Annu Rev Biochem. 2020;89:741-768.

4.	Whitfield C, Wear SS, Sande C. Assembly of Bacterial Capsular Polysaccharides and Exopolysaccharides. Annu Rev Microbiol. 2020;74:521-543.

5.	Ernst CM, Braxton JR, Rodriguez-Osorio CA, Zagieboylo AP, Li L, Pironti A, et al. Adaptive evolution of virulence and persistence in carbapenem-resistant Klebsiella pneumoniae. (vol 26, pg 705, 2020). Nat Med. 2020;26(8):1307-1307.
